# Prior emergency medical services utilization is a risk factor for in-hospital death among patients with substance misuse: a retrospective cohort study

**DOI:** 10.1186/s12873-024-01025-7

**Published:** 2024-07-09

**Authors:** Preeti Gupta, Anoop Mayampurath, Tim Gruenloh, Madeline Oguss, Askar Safipour Afshar, Michael Spigner, Megan Gussick, Matthew Churpek, Todd Lee, Majid Afshar

**Affiliations:** 1 Department of Biostatistics and Medical Informatics, School of Medicine and Public Health, University of Wisconsin-Madison, 610 Walnut Street, Madison, WI 53726 USA; 2 Department of Medicine, School of Medicine and Public Health, University of Wisconsin-Madison, 610 Walnut Street, Madison, WI 53726 USA; 3 Department of Emergency Medicine, School of Medicine and Public Health, University of Wisconsin-Madison, 600 Highland Avenue, Madison, WI 53792 USA

**Keywords:** Substance-Related Disorders, Emergency Medical Services, Hospital Mortality, Critical Illness, Data Linkage, Emergency Services Utilization, Facilities and Services Utilization, Health Services

## Abstract

**Background:**

Substance misuse poses a significant public health challenge, characterized by premature morbidity and mortality, and heightened healthcare utilization. While studies have demonstrated that previous hospitalizations and emergency department visits are associated with increased mortality in patients with substance misuse, it is unknown whether prior utilization of emergency medical service (EMS) is similarly associated with poor outcomes among this population. The objective of this study is to determine the association between EMS utilization in the 30 days before a hospitalization or emergency department visit and in-hospital outcomes among patients with substance misuse.

**Methods:**

We conducted a retrospective analysis of adult emergency department visits and hospitalizations (referred to as a hospital encounter) between 2017 and 2021 within the Substance Misuse Data Commons, which maintains electronic health records from substance misuse patients seen at two University of Wisconsin hospitals, linked with state agency, claims, and socioeconomic datasets. Using regression models, we examined the association between EMS use and the outcomes of in-hospital death, hospital length of stay, intensive care unit (ICU) admission, and critical illness events, defined by invasive mechanical ventilation or vasoactive drug administration. Models were adjusted for age, comorbidities, initial severity of illness, substance misuse type, and socioeconomic status.

**Results:**

Among 19,402 encounters, individuals with substance misuse who had at least one EMS incident within 30 days of a hospital encounter experienced a higher likelihood of in-hospital mortality (OR 1.52, 95% CI [1.05 – 2.14]) compared to those without prior EMS use, after adjusting for confounders. Using EMS in the 30 days prior to an encounter was associated with a small increase in hospital length of stay but was not associated with ICU admission or critical illness events.

**Conclusions:**

Individuals with substance misuse who have used EMS in the month preceding a hospital encounter are at an increased risk of in-hospital mortality. Enhanced monitoring of EMS users in this population could improve overall patient outcomes.

**Supplementary Information:**

The online version contains supplementary material available at 10.1186/s12873-024-01025-7.

## Background

Drug overdose-related mortality has been increasing in the United States for the past two decades [1]. Since 2019, mortality from drug overdoses and alcohol-related causes in the United States has risen approximately 10% per year, reaching an all-time high of over 100,000 premature deaths in 2021 [1, 2]. Stimulant and opioid-related deaths also continue to increase, with significant differences by race, ethnicity, and geographic region [3]. Substance misuse disorders are a significant driver of the global burden of disease, with alcohol use contributing to 99.2 million disability-adjusted life-years (DALYs) and drug use contributing to 31.8 million DALYs in 2016 [4]. Patients with these disorders have a higher risk for ICU admission than the general population, and these admissions account for 19–28% of all intensive care unit stays [5–8]. Despite being generally younger, these patients suffer from worse health system-related outcomes, more complications of illness, and higher mortality rates [5, 9]. These poor outcomes stem not only from direct consequences of substance misuse, such as drug overdose or alcohol withdrawal, but also from numerous other associated critical illnesses prevalent in these patients [9, 10, 11].

Several studies have reported that patients with substance misuse disproportionately utilize emergency medical services (EMS) compared to other patients [12–14]. For example, a recent study reported that approximately 40% of “super-frequent” EMS users (defined as 11 or more EMS transports to an emergency department in a year) were transported primarily for a substance misuse-related diagnosis [15]. EMS utilization attributable to substance misuse is likely even higher when accounting for the substantially increased risk of critical care conditions, such as organ dysfunction, infection, and metabolic derangements [9]. Previous studies have demonstrated that frequent emergency department (ED) visits and repeat hospitalizations are important risk factors for poor outcomes among patients with substance misuse [16]. However, EMS is usually the first and often the only healthcare contact during an encounter requiring medical assistance in patients with substance misuse. In fact, up to 42% of patients evaluated by EMS after an opioid-related incident refuse transport to the hospital [17, 18]. Thus, in addition to ED and hospital use, it is important to understand if prior utilization of EMS resources by patients with substance misuse is also a risk factor for poor health outcomes.

The primary objective of this study was to determine the association between in-hospital mortality and EMS use in the days leading up to a hospital admission or emergency visit. We hypothesize that EMS use in the 30 days before a hospital encounter increases the likelihood of in-hospital mortality after adjusting for confounders such as prior comorbidities, patient severity at admission, type of substance misuse, and socioeconomic status.

## Methods

### Data sources and study population

Data from the Substance Misuse Data Commons (SMDC) was used in this study [19]. The SMDC is a centralized repository of data from all adult (i.e., age ≥ 18 years) patients with an emergency department (ED) visit or inpatient hospitalization at two University of Wisconsin (UW) hospitals between 2008 and 2022 who had at least one substance use-related diagnosis code. The SMDC links electronic health records (EHR) with data from state agencies (e.g., EMS data from the Wisconsin Ambulance Run Data System, Vital Statistics), a national mortality data source, medical and pharmacy claims, prescription drug monitoring systems, and socioeconomic indicators. We conducted a retrospective, observational analysis of ED visits or hospitalizations in the SMDC between 2017 and 2021 because this corresponded to the years with complete EHR, EMS, and claims data available. To avoid confounding from recent illnesses, we excluded patients who had a recorded ED visit or hospitalization, either in our health system’s EHR or in the statewide hospital claims data, within the prior 30 days of the index encounter. All EMS data were derived from a statewide EMS database, the Wisconsin Ambulance Run Data System, that covers over 90% of the ambulance services in the state of Wisconsin. Supplementary Fig. A describes a flowchart of the cohort selection process. The study was approved by the UW Internal Review Board (IRB# 2021–0553), which did not require explicit consent from the participants.

### Outcomes

The primary outcome of interest was in-hospital mortality, recorded in the EHR. We also considered secondary outcomes of hospital length of stay, ICU admission, and critical illness events, defined as requiring invasive mechanical ventilation or vasoactive drug administration during the hospitalization. Ventilator or vasopressor use that occurred during surgical procedures was not considered part of the definition of critical illness events.

### Exposures and confounders

Our primary exposure was the occurrence of at least one EMS incident in the 30 days prior to each hospital encounter. We excluded the EMS incident that brought the patient to the hospital by excluding any incidents within 24 hours before hospital admission, as we considered them part of the deterioration that led to a hospital encounter. The remaining EMS incidents underwent binary categorization of 0 vs ≥ 1 EMS incidents, based on our analysis that showed only one percent of encounters were preceded by more than one EMS incident in the prior 1–30 days (0 EMS visit: *n* = 18,411 [95%], 1 EMS visit: *n* = 742 [4%], > 1 EMS Visit: *n* = 249 [1%]).

We adjusted for age, sex, prior comorbidities, severity of illness at presentation, type of substance misuse, and socioeconomic status. Prior comorbidities were assessed using Elixhauser comorbidities from all prior admissions [20]. We used the first Modified Early Warning Score (MEWS) as an indicator for patient severity at presentation, stratified into low (0–1), medium (2–4), and high (5–14) risk [21]. Type of substance misuse was identified by International Classification of Disease codes for all types of substance misuse, urine cocaine toxicology, and serum alcohol levels. Socioeconomic status was estimated using the neighborhood area deprivation index (ADI) state rankings derived from the patient’s home address. The state ADI is a multidimensional score, between 1 and 10, used to characterize the socioeconomic conditions of a census block group, with higher numbers representing more disadvantaged neighborhoods. This tool has been previously described and validated for several health outcomes [22]. Missing values for confounders (see Supplementary Table A), were imputed using the sample median. We also analyzed our dataset using the multiple imputation method as an alternative to median imputation.

### Statistical analysis

We compared the characteristics of patients who survived to discharge to those who did not using Chi-squared tests for categorical variables and Student’s t-test or Wilcoxon Rank Sum test for continuous variables. The same tests were used to compare the characteristics of patients who had a prior 30-day EMS incident against those who did not. We used logistic regression to assess the association between the occurrence of an EMS incident in the 30 days prior to an encounter and the primary outcome, with sequential adjustment of confounders. Variance inflation factor analysis was performed to test variables for multicollinearity. The secondary outcome of hospital length of stay was analyzed with multivariable linear regression with a log-transformed outcome, and multivariable logistic regression was used to analyze ICU admission and critical illness events. We followed the STROBE criteria for cohort studies (Supplementary Table B) [23]. All analyses were performed in RStudio using R version 4.3.1 (R Project for Statistical Computing, Vienna, Austria). Two-sided *P* < 0.05 and 95% confidence intervals were utilized to indicate statistical significance.

## Results

There were 28,243 hospital encounters between 2017 and 2021 within the SMDC. After excluding 4,214 encounters with ED visits or hospitalizations at UW hospital in the previous 30 days and then excluding 4,627 encounters with ED visits or hospitalizations in the claims data, the total cohort consisted of 19,402 encounters. The majority of encounters (*n* = 12,156, 62.7%) were for male patients (see Table 1). The distribution of substance misuse among patients in our cohort was: alcohol-only (*n* = 10,264, 52.9%), opioid-only misuse (*n* = 2,689, 13.9%), other single substance (*n* = 2,036, 10.5%), and polysubstance (*n* = 4,413, 22.7%). The primary outcome of in-hospital death occurred during 427 (0.02%) encounters and those who died were older (mean age 56 years vs. 47 years) than those who survived. Analysis of the most frequently occurring prior comorbidities demonstrated that patients who died during their hospitalization had a greater prevalence of liver disease prior to the hospitalization (36.5% vs. 29.0%) and lower rates of depression (36.1% vs. 52.8%). They were also more likely to have a high MEWS on presentation (40.0% vs. 7.1%), were more likely to have alcohol-only misuse (60.9% vs. 52.7%) and came from more disadvantaged neighborhoods (median ADI 5 vs. 3) than those who survived the hospitalization.

**Table 1:** Characteristics of the patients in the study cohort, stratified by primary outcome

	Survived to Discharge (*n* = 18,975)	Died in Hospital (*n* = 427)	*P*-value
Age (mean (SD))	46.80 (17.00)	56.05 (15.02)	< 0.001
Male (%)	11889 (62.7)	267 (62.5)	0.998
Cardiac Arrhythmias^a^ (%)	8226 (43.4)	173 (40.5)	0.26
Hypertension, uncomplicated^a^ (%)	8128 (42.8)	202 (47.3)	0.07
Chronic Pulmonary Disease^a^ (%)	6438 (33.9)	140 (32.8)	0.66
Liver Disease^a^ (%)	5512 (29.0)	156 (36.5)	0.001
Fluid and Electrolyte Disorders^a^ (%)	7457 (39.3)	174 (40.7)	0.58
Depression^a^ (%)	10017 (52.8)	154 (36.1)	< 0.001
MEWS Score^b^ (%)			< 0.001
Low (0–1)	8752 (46.1)	61 (14.3)	
Medium (2–4)	8867 (46.7)	195 (45.7)	
High (5–14)	1356 (7.1)	171 (40.0)	
Type of Substance Misuse (%)			< 0.001
Alcohol Only	10004 (52.7)	260 (60.9)	
Non-Alcohol, Non-Opioid^c^	2022 (10.7)	14 (3.3)	
Opioid only	2625 (13.8)	64 (15.0)	
Polysubstance	4324 (22.8)	89 (20.8)	
ADI^d^ State Rank (median [IQR])	3.00 [2.00, 6.00]	5.00 [2.00, 7.00]	< 0.001
ICU^e^ Admission (%)	2335 (12.3)	338 (79.2)	< 0.001
Ventilator or Vasopressors (%)	1920 (10.1)	329 (77.0)	< 0.001
Hospital Length of Stay (median [IQR])	1.83 [0.22, 4.74]	5.19 [2.18, 11.68]	< 0.001

^a^The most frequently occurring Elixhauser co-morbidities are shown here

^b^MEWS = Modified Early Warning Score

^c^Non-alcohol, non-opioid drugs include cocaine, benzodiazepines, amphetamines, cannabis, and barbiturates

^d^ADI = Area Deprivation Index

^e^ICU = Intensive Care Unit

In the 30 days prior to hospitalization, 18,416 (94.9%) encounters did not have a prior EMS incident, while 986 (5.1%) had at least one EMS incident (Table 2). Compared to those without a prior EMS visit, patients with prior 30-day EMS incidents were older (mean age 54 years vs. 47 years), more likely to have pre-existing comorbidities, and came from more disadvantaged neighborhoods (median ADI 4 vs. 3). No differences were observed in the initial MEWS.

**Table 2:** Characteristics of the patients in the study cohort, stratified by EMS utilization in the 1–30 days prior to the hospital encounter

	No prior EMS incidents (*n* = 18,416)	Prior EMS incidents (*n* = 986)	*P*-value
Age (mean (SD))	46.63 (16.98)	53.91 (16.24)	< 0.001
Male (%)	11565 (62.8)	591 (59.9)	0.08
Cardiac Arrhythmias^a^ (%)	7838 (42.6)	561 (56.9)	< 0.001
Hypertension, uncomplicated^a^ (%)	7787 (42.3)	543 (55.1)	< 0.001
Chronic Pulmonary Disease^a^ (%)	6156 (33.4)	422 (42.8)	< 0.001
Liver Disease^a^ (%)	5310 (28.8)	358 (36.3)	< 0.001
Fluid and Electrolyte Disorders^a^ (%)	7101 (38.6)	530 (53.8)	< 0.001
Depression^a^ (%)	9616 (52.2)	555 (56.3)	0.01
MEWS Score^b^ (%)			0.002
Low (0–1)	1437 (7.8)	90 (9.1)	
Medium (2–4)	8418 (45.7)	395 (40.1)	
High (5–14)	8561 (46.5)	501 (50.8)	
Type of Substance Misuse (%)			< 0.001
Alcohol Only	9772 (53.1)	492 (49.9)	
Non-Alcohol, Non-Opioid^c^	1942 (10.5)	94 (9.5)	
Opioid only	2492 (13.5)	197 (20.0)	
Polysubstance	4210 (22.9)	203 (20.6)	
ADI^d^ State Rank (median [IQR])	3.00 [2.00, 6.00]	4.00 [2.00, 6.00]	< 0.001
In-Hospital Death (%)	387 (2.1)	40 (4.1)	< 0.001
ICU^e^ Admission (%)	2513 (13.6)	160 (16.2)	0.05
Ventilator or Vasopressors (%)	2098 (11.4)	151 (15.3)	< 0.001
Hospital Length of Stay (median days [IQR])	1.82 [0.22, 4.71]	3.58 [0.92, 8.59]	< 0.001

^a^The most frequently occurring Elixhauser co-morbidities are shown here

^b^MEWS = Modified Early Warning Score

^c^Non-alcohol, non-opioid drugs include cocaine, benzodiazepines, amphetamines, cannabis, and barbiturates

^d^ADI = Area Deprivation Index

^e^ICU = Intensive Care Unit

The association between the occurrence of EMS incidents in the 30 days prior to an encounter and risk of in-hospital mortality as odds ratios (OR) is depicted in Table 3. In the unadjusted model, an EMS incident in the prior 30 days increased the odds of in-hospital mortality by 97%. After adjusting for patient age, sex, and prior comorbidities, this association was attenuated, with further adjustments for initial MEWS, a marker of risk for deterioration on presentation to the hospital, slightly decreasing the association. Type of substance misuse did not impact the estimates of association while adjustments for the state ADI ranking, a proxy for socioeconomic status, resulted in a slight reduction in the association. In the fully-adjusted model, there remained an increased risk of in-hospital mortality among patients with EMS incidents prior to their hospitalization compared to those who had not used EMS (OR 1.52 95% CI [1.05 – 2.14]). Similar results were observed when considering a multiple imputation method instead of imputing using medians (OR 1.47 95% CI [1.04 – 2.10]). Supplementary Table C depicts the ORs for each variable in the fully adjusted in-hospital mortality model and shows age, liver disease, metastatic cancer, coagulopathy, initial MEWS, alcohol-only misuse, polysubstance misuse, and ADI ranking were also significantly associated with increased risk of in-hospital death. No multicollinearity was detected, with all variance inflation factors less than 2.5 [24].

**Table 3:** Unadjusted and sequentially adjusted Odds Ratios (OR) of in-hospital mortality with EMS utilization in the prior 1–30 days

Sequential Adjustments of Multivariable Models	In-Hospital Mortality, OR (95% CI)
Unadjusted	1.97 (1.39—2.71)
+ Age, Sex, and Elixhauser Comorbidities	1.65 (1.15—2.28)
+ MEWS Score^a^	1.58 (1.09 – 2.22)
+ Type of Substance	1.58 (1.10 – 2.23)
+ State ADI^b^	1.52 (1.05 – 2.14)

^a^MEWS = Modified Early Warning Score

^b^ADI = Area Deprivation Index

The adjusted associations between prior EMS incidents and the secondary outcomes are shown in Table 4. Hospital length of stay increased by 39% [95% CI, 25%—53%] in encounters with a prior EMS incident. The adjusted analysis did not demonstrate an association between the occurrence of EMS incidents before the hospitalization and ICU admission during the hospitalization (OR, 0.94 [95% CI, 0.77 – 1.15). Exposure to EMS incidents before an encounter was also not associated with a requirement for invasive mechanical ventilation or vasopressors during the hospitalization, after adjusting for confounding variables (OR, 1.14 [95% CI, 0.93 – 1.39). Supplementary Tables D, E, and F depict the ORs for each of the model’s input variables and each of the secondary outcomes.

**Table 4:** Fully adjusted association between EMS incidents in the prior 1–30 days and the secondary outcomes of ICU admission, critical illness events and hospital length of stay

Secondary Outcome	Association with EMS Utilization
Hospital Length of Stay, % increase (95% CI)	39 (25 –53)
ICU^a^ Admission, OR^b^ (95% CI)	0.94 (0.77 – 1.15)
Ventilator or Vasopressors, OR^b^ (95% CI)	1.14 (0.93 – 1.39)

^a^ICU = Intensive Care Unit

^b^OR = Odds Ratio

## Discussion

This study used a comprehensively linked dataset across EHR, EMS, and claims data sources to determine the association between EMS utilization and subsequent in-hospital outcomes among individuals with substance misuse. Our results indicate that individuals who utilized EMS in the month prior to a hospital encounter had a higher likelihood of in-hospital mortality compared to those without EMS use in the preceding month. The association persisted after adjusting for potential confounders. While no significant association was observed between prior 30-day EMS incidents and ICU admission or critical illness after adjusting for confounders, patients with the primary exposure were more likely to experience a slightly longer hospital length of stay. Our study indicates that prior EMS users are a distinct cohort of substance misuse patients with a higher risk of mortality and thus could benefit from closer monitoring or timely interventions.

Prior studies have examined increased acute care utilization as a risk factor for mortality in patients with substance misuse [16]. Patients who visited the ED 1–3 times in the prior year for any medical reason had almost double the odds of mortality compared to those who did not [16]. In the same study, hospitalization in the previous year was associated with more than a ninefold risk of death. Our study adds to this body of scientific research by examining the association between past EMS use and in-hospital outcomes. We found that an EMS visit in the 30 days prior to an ED visit or hospital admission is associated with a 50% increased risk of experiencing in-hospital mortality. Given that many patients with substance misuse refuse transport to the hospital [17, 18], our results support the development of interventions in the prehospital space to prevent poor 30-day outcomes. For example, patients could benefit from tertiary prevention efforts by EMS, including initiation of treatments like buprenorphine and linkage to ongoing care for patients with opioid misuse.

Given that a significant proportion of EMS use is by individuals with substance misuse, it is crucial to understand how EMS usage patterns are linked with outcomes [9, 13–15]. Unfortunately, EMS data is frequently disaggregated from hospital data, making it challenging to incorporate in risk models for critical outcomes. Our study is the first to use granular data harmonized across in-hospital and out-of-hospital settings, thereby providing this key analysis. Furthermore, we were able to effectively isolate statewide EMS use as an exposure by using both EHR and claims data to exclude prior ED visits and hospitalizations.

Studies have consistently found that EMS users have a higher burden of chronic conditions compared to non-users [25–28]. In our data, we observed that patients who had an EMS incident had higher rates of hypertension, chronic pulmonary disease, liver disease, fluid and electrolyte disorders, cardiac arrhythmias, and depression than those who did not interact with EMS. Importantly, in other studies examining EMS use across medical conditions such as ST-elevation myocardial infarction, EMS users still experienced worse outcomes after adjusting for these comorbidities and several other relevant factors [25]. In another example, EMS users transported in septic shock presented with higher Charlson comorbidity scores and worse initial vital signs than non-users [26]. Even after adjusting for these factors in the data analysis, they still experienced higher mortality, suggesting other contributing factors remain.

Several studies have also demonstrated an association between EMS utilization and markers of socioeconomic disadvantage, such as income, level of educational attainment, and type of health insurance [28–33]. In our study, patients with prior EMS incidents lived in neighborhoods with greater socioeconomic disadvantage, as estimated using the ADI. Furthermore, in patients with substance misuse, lower socioeconomic status has been associated with worse outcomes [34–38]. Counties with smaller changes in median household income growth, greater rises in unemployment rates, and greater increases in vacant housing experienced increased drug mortality rates compared to counties that did not experience these negative socioeconomic effects [38]. Our study aligns with these prior findings, with our cohort demonstrating an association between mortality and the state area deprivation index. However, even after adjusting for socioeconomic status, the positive association remained between EMS use and in-hospital death.

After adjusting for potential confounders, a 30-day prior EMS visit was also associated with a 39% increase in hospital length of stay. Considering the median length of stay of 2 days for patients who did not have a 30-day prior EMS, this would imply an increase by three-fourths of a day for patients with a 30-day prior EMS. This minor increase is unlikely to be clinically significant. Although patients with a 30-day prior EMS incident were observed to have higher rates of ICU admission and critical illness events in the unadjusted analysis, we did not notice a statistically significant association between these outcomes and 30-day prior EMS in the fully-adjusted model. It is possible that patients with substance misuse uniformly experience critical care interventions, such as transferring to the ICU or being mechanically ventilated, based on current illness and not distal risk factors such as prior 30-day EMS visits. Additionally, our analysis may have missed residual confounding for determining these associations accurately. The mismatch between the positive association to in-hospital mortality and the lack of statistically significant association to secondary outcomes needs further investigation in a larger population.

Limitations of this study include the possibility of residual confounding from using retrospective and observational data. Additionally, we sought to isolate EMS use as an exposure, but it is possible that patients with prior EMS incidents may have additionally had prior hospitalizations at other hospitals in the past month. To attenuate this, we used claims data to capture hospitalizations that were not available in our EHR, which is a notable improvement over prior studies that account for events using either the EHR or claims data. While we strove to isolate the effect of EMS, our exclusion criteria could add complexity to risk stratification in practice. The retrospective design also resulted in some missing AVPU (Alert, Voice, Pain, Responsive) values that were part of the MEWS and ADI values, and this missingness could have a systematic relationship with our exposure and outcomes. Additionally, though the exposure was comprehensively measured statewide, the study outcomes were measured at a single medical center and could be subject to institutional practices. The single-center dataset also limited our sample size and ability to evaluate prior EMS incidents in a dose-dependent way. Even though we linked to a statewide database of EMS encounters and statewide hospital claims, our sample size was limited to our single-center EHR data with respect to prior EMS incidents, which limited our ability to stratify by mode of EMS transport or evaluate initial EMS impression as a potential risk factor. Future prospective studies can include additional centers and examine specific risk factors of EMS incidents in order to isolate and understand the association between EMS incidents and hospital mortality more thoroughly. Lastly, we measured a limited number of outcomes. Assessing more granular outcomes, such as organ dysfunction, may better illuminate the pathway to mortality.

## Conclusions

In conclusion, our data demonstrate that EMS use is associated with increased 30-day in-hospital mortality among patients with substance misuse. This finding suggests that this setting may represent an opportunity for the delivery of timely interventions aimed at alleviating the burden of disease related to this disorder. Additionally, it indicates that future efforts to risk stratify patients with substance misuse may benefit from incorporating EMS utilization patterns. Lastly, our work reinforces the need for healthcare data connectedness to fully appreciate the continuum of possible care pathways that may be relevant to informing future policy.

## Supplementary Information

Supplementary Material 1.

## Data Availability

Data availability is subject to existing data use agreements. For further details, please contact the corresponding author.

## Abbreviations

ADI: Area Deprivation Index
DALYs: Disability-Adjusted Life-Years
ED: Emergency Department
EHR: Electronic Health Records
EMS: Emergency Medical Services
ICU: Intensive Care Unit
IRB: Institutional Review Board
MEWS: Modified Early Warning Score
OR: Odds Ratio
SMDC: Substance Misuse Data Commons
UW: University of Wisconsin
