# Correction: Prior emergency medical services utilization is a risk factor for in-hospital death among patients with substance misuse: a retrospective cohort study

**DOI:** 10.1186/s12873-024-01109-4

**Published:** 2024-10-11

**Authors:** Preeti Gupta, Anoop Mayampurath, Tim Gruenloh, Madeline Oguss, Askar Safipour Afshar, Michael Spigner, Megan Gussick, Matthew Churpek, Todd Lee, Majid Afshar

**Affiliations:** 1 Division of Pulmonary, Critical Care, Sleep and Allergy, University of Illinois Chicago, 840 South Wood Street, Chicago, IL 60612 USA; 2 Department of Medicine, School of Medicine and Public Health, University of Wisconsin-Madison, 610 Walnut Street, Madison, WI 53726 USA; 3 Department of Biostatistics and Medical Informatics, School of Medicine and Public Health, University of Wisconsin-Madison, 610 Walnut Street, Madison, WI 53726 USA; 4 Department of Emergency Medicine, School of Medicine and Public Health, University of Wisconsin- Madison, 600 Highland Avenue, Madison, WI 53792 USA; 5 College of Pharmacy, University of Illinois Chicago, 833 S. Wood Street, Chicago, IL 60612 USA

**Correction: BMC Emergency Medicine (2024) 24:110**

10.1186/s12873-024-01025-7

The original article contains erroneous affiliations for co-authors, Preeti Gupta and Todd Lee; the corrected affiliations can be viewed in the authorship and backmatter of this Correction article.

1. Division of Pulmonary, Critical Care, Sleep and Allergy, University of Illinois Chicago, 840 South Wood Street, Chicago, Illinois 60612, USA
2. Department of Medicine, School of Medicine and Public Health, University of Wisconsin-Madison, 610 Walnut Street, Madison, WI 53726, USA
3. Department of Biostatistics and Medical Informatics, School of Medicine and Public Health, University of Wisconsin-Madison, 610 Walnut Street, Madison, WI 53726, USA
4. Department of Emergency Medicine, School of Medicine and Public Health, University of Wisconsin-Madison, 600 Highland Avenue, Madison, WI 53792, USA
5. College of Pharmacy, University of Illinois Chicago, 833 S. Wood Street, Chicago, IL 60612, USA

