# Supplementary material for: Prior emergency medical services utilization is a risk factor for in-hospital death among patients with substance misuse: a retrospective cohort study
Source: BMC Emerg Med. 2024 Jul 9;24:110. doi: 10.1186/s12873-024-01025-7 (PMC11234660; doi:10.1186/s12873-024-01025-7)
Supplement: Supplementary file 1 — Supplementary Material 1. [file 12873_2024_1025_MOESM1_ESM.docx]

**SUPPLEMENTARY FIGURES AND TABLES**

Supplementary Figure A. Flowchart of patient selection.

**
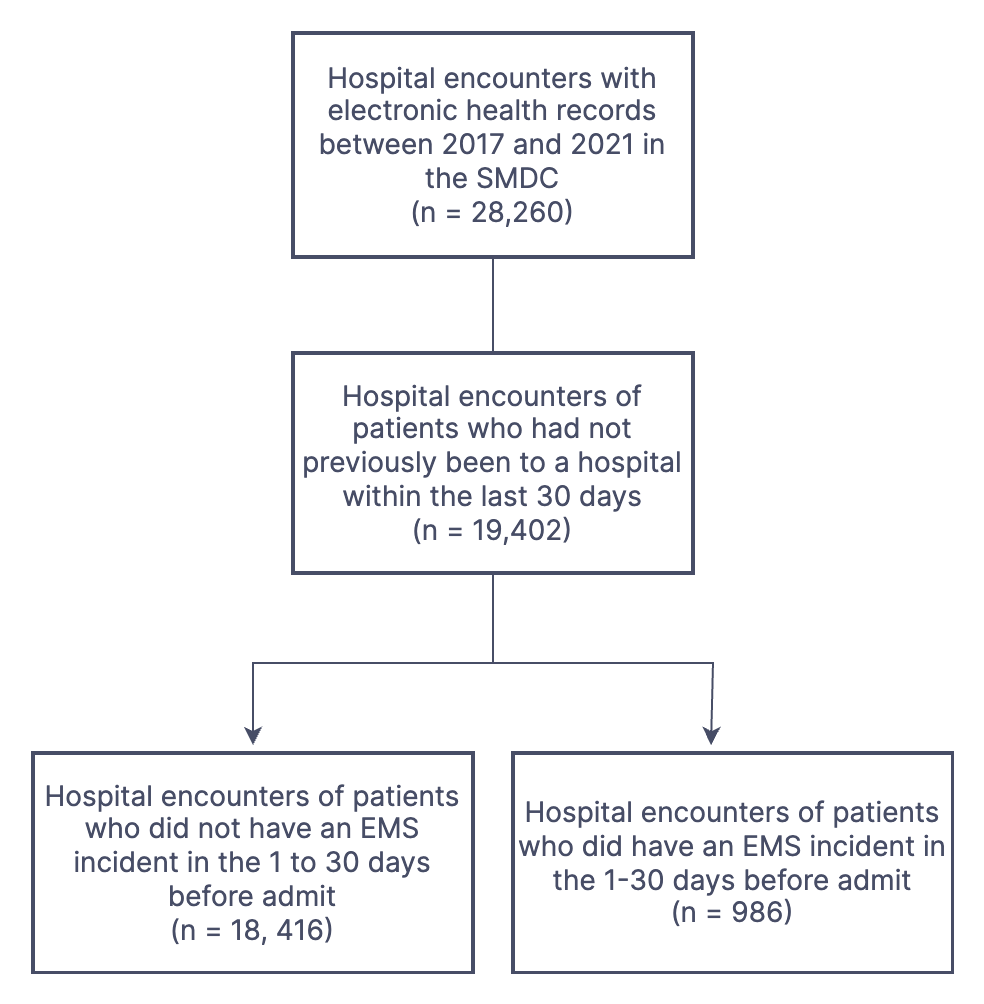
**

Supplementary Table A. Missingness of Imputed Variables

| **Variable** | **Number of Encounters with Missing Data (Percent)** | **Number of Encounters without EMS in the prior 1-30 days with Missing Data (Percent)** | **Number of Encounters with EMS in the prior 1-30 days with Missing Data (Percent)** |
| --- | --- | --- | --- |
| State ADI^a^ | 848 (4.4%) | 788 (4.3%) | 60 (6.1%) |
| AVPU^b^ (part of MEWS^c^) | 3,127 (16.1%) | 3,036 (16.5%) | 91 (9.8%) |
| MEWS | 9 (<0.1%) | 9 (<0.1%) | 0 (0%) |

^a^ADI = Area Deprivation Index; ^b^AVPU = Alert, Voice, Pain, Unresponsive; ^c^MEWS = Modified Early Warning Score

Supplementary Table B. STROBE Statement—Checklist of items

|  | Item No | Recommendation | Page  # |
| --- | --- | --- | --- |
| **Title and abstract** | 1 | (*a*) Indicate the study’s design with a commonly used term in the title or the abstract | 1 |
|  |  | (*b*) Provide in the abstract an informative and balanced summary of what was done and what was found | 2-3 |
| Introduction | | |  |
| Background/rationale | 2 | Explain the scientific background and rationale for the investigation being reported | 5-6 |
| Objectives | 3 | State specific objectives, including any prespecified hypotheses | 6 |
| Methods | | |  |
| Study design | 4 | Present key elements of study design early in the paper | 6 |
| Setting | 5 | Describe the setting, locations, and relevant dates, including periods of recruitment, exposure, follow-up, and data collection | 6-8 |
| Participants | 6 | (*a*) Give the eligibility criteria, and the sources and methods of selection of participants. Describe methods of follow-up | 6-7 |
|  |  | (*b*) For matched studies, give matching criteria and number of exposed and unexposed | N/A |
| Variables | 7 | Clearly define all outcomes, exposures, predictors, potential confounders, and effect modifiers. Give diagnostic criteria, if applicable | 7-8 |
| Data sources/ measurement | 8* | For each variable of interest, give sources of data and details of methods of assessment (measurement). Describe comparability of assessment methods if there is more than one group | 6-8 |
| Bias | 9 | Describe any efforts to address potential sources of bias | 8 |
| Study size | 10 | Explain how the study size was arrived at | N/A |
| Quantitative variables | 11 | Explain how quantitative variables were handled in the analyses. If applicable, describe which groupings were chosen and why | 7-8 |
| Statistical methods | 12 | (*a*) Describe all statistical methods, including those used to control for confounding | 8-9 |
|  |  | (*b*) Describe any methods used to examine subgroups and interactions | N/A |
|  |  | (*c*) Explain how missing data were addressed | 8, Supp. Table A |
|  |  | (*d*) If applicable, explain how loss to follow-up was addressed | N/A |
|  |  | (*e*) Describe any sensitivity analyses | 8 |
| Results | | |  |
| Participants | 13* | (a) Report numbers of individuals at each stage of study—eg numbers potentially eligible, examined for eligibility, confirmed eligible, included in the study, completing follow-up, and analysed | 9 |
|  |  | (b) Give reasons for non-participation at each stage | N/A |
|  |  | (c) Consider use of a flow diagram | Supp. Figure A |
| Descriptive data | 14* | (a) Give characteristics of study participants (eg demographic, clinical, social) and information on exposures and potential confounders | 9-10, Table 1 and 2 |
|  |  | (b) Indicate number of participants with missing data for each variable of interest | Supp. Table A |
|  |  | (c) Summarise follow-up time (eg, average and total amount) | N/A |
| Outcome data | 15* | Report numbers of outcome events or summary measures over time | 9, Table 1 and 2 |
| Main results | 16 | (*a*) Give unadjusted estimates and, if applicable, confounder-adjusted estimates and their precision (eg, 95% confidence interval). Make clear which confounders were adjusted for and why they were included | 12-13, Table 3 and 4 |
|  |  | (*b*) Report category boundaries when continuous variables were categorized | Table 1 and 2 |
|  |  | (*c*) If relevant, consider translating estimates of relative risk into absolute risk for a meaningful time period | N/A |
| Other analyses | 17 | Report other analyses done—eg analyses of subgroups and interactions, and sensitivity analyses | 12 |
| Discussion | | |  |
| Key results | 18 | Summarise key results with reference to study objectives | 13-14 |
| Limitations | 19 | Discuss limitations of the study, taking into account sources of potential bias or imprecision. Discuss both direction and magnitude of any potential bias | 16-17 |
| Interpretation | 20 | Give a cautious overall interpretation of results considering objectives, limitations, multiplicity of analyses, results from similar studies, and other relevant evidence | 13-17 |
| Generalisability | 21 | Discuss the generalisability (external validity) of the study results | 16-17 |
| Other information | | |  |
| Funding | 22 | Give the source of funding and the role of the funders for the present study and, if applicable, for the original study on which the present article is based | 19 |

Supplementary Table C. Fully Adjusted Model for In-Hospital Death

|  | Odds Ratio | 2.5% | 97.5% | P-value |
| --- | --- | --- | --- | --- |
| >=1 EMS visit | 1.52 | 1.05 | 2.14 | 0.02 |
| Age | 1.04 | 1.03 | 1.05 | <0.001 |
| Female | 1.18 | 0.95 | 1.47 | 0.14 |
| Congestive heart failure | 1.28 | 0.89 | 1.83 | 0.17 |
| Cardiac arrhythmias | 0.78 | 0.59 | 1.03 | 0.08 |
| Valvular disease | 1.02 | 0.73 | 1.43 | 0.89 |
| Pulmonary circulation disorders | 0.89 | 0.61 | 1.28 | 0.55 |
| Peripheral vascular disorders | 0.92 | 0.66 | 1.25 | 0.58 |
| Hypertension, uncomplicated; | 0.77 | 0.59 | 1.01 | 0.06 |
| Hypertension, complicated | 1.05 | 0.70 | 1.56 | 0.81 |
| Paralysis | 0.87 | 0.43 | 1.59 | 0.68 |
| Other neurological disorders | 0.88 | 0.66 | 1.17 | 0.38 |
| Chronic pulmonary disease | 0.91 | 0.71 | 1.17 | 0.47 |
| Diabetes, uncomplicated | 1.21 | 0.82 | 1.77 | 0.33 |
| Diabetes, complicated | 0.86 | 0.55 | 1.36 | 0.53 |
| Hypothyroidism | 0.88 | 0.61 | 1.25 | 0.48 |
| Renal failure | 1.34 | 0.92 | 1.93 | 0.12 |
| Liver disease | 1.52 | 1.18 | 1.96 | 0.001 |
| Peptic ulcer disease, excluding bleeding | 0.81 | 0.53 | 1.22 | 0.33 |
| AIDS/HIV | 0.73 | 0.12 | 2.38 | 0.66 |
| Lymphoma | 1.42 | 0.67 | 2.70 | 0.31 |
| Metastatic cancer | 2.44 | 1.47 | 4.03 | 0.001 |
| Solid tumor, without metastasis | 0.61 | 0.40 | 0.91 | 0.02 |
| Rheumatoid arthritis/collagen vascular disease | 1.00 | 0.67 | 1.44 | 0.98 |
| Coagulopathy | 1.50 | 1.10 | 2.02 | 0.009 |
| Obesity | 0.76 | 0.56 | 1.02 | 0.07 |
| Weight loss | 1.05 | 0.79 | 1.38 | 0.76 |
| Fluid and electrolyte disorders | 0.96 | 0.71 | 1.29 | 0.78 |
| Blood loss anemia | 1.42 | 0.91 | 2.18 | 0.12 |
| Deficiency anemia | 0.98 | 0.70 | 1.36 | 0.92 |
| Psychoses | 0.77 | 0.49 | 1.16 | 0.23 |
| Depression | 0.58 | 0.45 | 0.74 | <0.001 |
| MEWS Score^a^ | 1.72 | 1.64 | 1.80 | <0.001 |
| Alcohol only | 2.00 | 1.19 | 3.65 | 0.02 |
| Opioid only | 1.81 | 1.01 | 3.44 | 0.06 |
| Polysubstance | 2.12 | 1.22 | 3.94 | 0.01 |
| ADI State Rank^b^ | 1.09 | 1.05 | 1.14 | <0.001 |

^a^MEWS = Modified Early Warning Score; ^b^ADI = Area Deprivation Index;

Supplementary Table D. Fully Adjusted Model for ICU Admission

|  | Odds Ratio | 2.5% | 97.5% | P-value |
| --- | --- | --- | --- | --- |
| >=1 EMS visit | 0.94 | 0.77 | 1.15 | 0.56 |
| Age | 1.03 | 1.02 | 1.03 | <0.001 |
| Female | 0.74 | 0.67 | 0.82 | <0.001 |
| Congestive heart failure | 1.06 | 0.89 | 1.26 | 0.54 |
| Cardiac arrhythmias | 0.71 | 0.63 | 0.81 | <0.001 |
| Valvular disease | 1.31 | 1.12 | 1.54 | 0.001 |
| Pulmonary circulation disorders | 1.28 | 1.07 | 1.52 | 0.006 |
| Peripheral vascular disorders | 1.17 | 1.01 | 1.36 | 0.04 |
| Hypertension, uncomplicated; | 0.98 | 0.86 | 1.11 | 0.71 |
| Hypertension, complicated | 0.81 | 0.66 | 0.99 | 0.04 |
| Paralysis | 0.93 | 0.69 | 1.23 | 0.60 |
| Other neurological disorders | 1.22 | 1.07 | 1.39 | 0.003 |
| Chronic pulmonary disease | 1.00 | 0.90 | 1.12 | 0.94 |
| Diabetes, uncomplicated | 1.22 | 1.01 | 1.46 | 0.04 |
| Diabetes, complicated | 0.80 | 0.64 | 0.99 | 0.04 |
| Hypothyroidism | 1.03 | 0.87 | 1.22 | 0.70 |
| Renal failure | 1.14 | 0.94 | 1.37 | 0.17 |
| Liver disease | 1.23 | 1.09 | 1.39 | 0.001 |
| Peptic ulcer disease, excluding bleeding | 0.89 | 0.73 | 1.08 | 0.25 |
| AIDS/HIV | 0.69 | 0.37 | 1.17 | 0.19 |
| Lymphoma | 1.03 | 0.69 | 1.50 | 0.89 |
| Metastatic cancer | 0.69 | 0.53 | 0.90 | 0.007 |
| Solid tumor, without metastasis | 1.03 | 0.87 | 1.22 | 0.75 |
| Rheumatoid arthritis/collagen vascular disease | 0.87 | 0.72 | 1.05 | 0.14 |
| Coagulopathy | 1.09 | 0.93 | 1.26 | 0.28 |
| Obesity | 0.92 | 0.81 | 1.05 | 0.24 |
| Weight loss | 0.80 | 0.70 | 0.92 | 0.002 |
| Fluid and electrolyte disorders | 0.81 | 0.71 | 0.92 | 0.001 |
| Blood loss anemia | 1.18 | 0.93 | 1.49 | 0.17 |
| Deficiency anemia | 0.81 | 0.69 | 0.96 | 0.02 |
| Psychoses | 0.66 | 0.54 | 0.79 | <0.001 |
| Depression | 0.68 | 0.61 | 0.76 | <0.001 |
| MEWS Score^a^ | 1.67 | 1.62 | 1.71 | <0.001 |
| Alcohol only | 1.44 | 1.20 | 1.74 | <0.001 |
| Opioid only | 1.10 | 0.88 | 1.38 | 0.39 |
| Polysubstance | 1.49 | 1.22 | 1.82 | <0.001 |
| ADI State Rank^b^ | 1.13 | 1.11 | 1.15 | <0.001 |

^a^MEWS = Modified Early Warning Score; ^b^ADI = Area Deprivation Index;

Supplementary Table E. Fully Adjusted Model for Invasive Mechanical Ventilation or Vasopressor Requirement

|  | Odds Ratio | 2.5% | 97.5% | P-value |
| --- | --- | --- | --- | --- |
| >=1 EMS visit | 1.14 | 0.93 | 1.39 | 0.21 |
| Age | 1.02 | 1.02 | 1.03 | <0.001 |
| Female | 0.81 | 0.73 | 0.91 | <0.001 |
| Congestive heart failure | 1.09 | 0.91 | 1.31 | 0.33 |
| Cardiac arrhythmias | 0.68 | 0.60 | 0.78 | <0.001 |
| Valvular disease | 1.35 | 1.14 | 1.59 | <0.001 |
| Pulmonary circulation disorders | 1.21 | 1.01 | 1.44 | 0.04 |
| Peripheral vascular disorders | 1.33 | 1.14 | 1.55 | <0.001 |
| Hypertension, uncomplicated; | 0.92 | 0.81 | 1.05 | 0.23 |
| Hypertension, complicated | 0.80 | 0.65 | 0.98 | 0.03 |
| Paralysis | 0.89 | 0.65 | 1.19 | 0.45 |
| Other neurological disorders | 1.30 | 1.14 | 1.49 | <0.001 |
| Chronic pulmonary disease | 1.01 | 0.90 | 1.14 | 0.87 |
| Diabetes, uncomplicated | 1.05 | 0.86 | 1.28 | 0.62 |
| Diabetes, complicated | 0.94 | 0.75 | 1.18 | 0.59 |
| Hypothyroidism | 0.93 | 0.78 | 1.11 | 0.43 |
| Renal failure | 1.35 | 1.11 | 1.63 | 0.002 |
| Liver disease | 1.21 | 1.06 | 1.37 | 0.004 |
| Peptic ulcer disease, excluding bleeding | 1.00 | 0.82 | 1.22 | 0.98 |
| AIDS/HIV | 0.53 | 0.26 | 0.99 | 0.07 |
| Lymphoma | 1.06 | 0.69 | 1.56 | 0.79 |
| Metastatic cancer | 0.81 | 0.61 | 1.07 | 0.15 |
| Solid tumor, without metastasis | 0.97 | 0.80 | 1.16 | 0.71 |
| Rheumatoid arthritis/collagen vascular disease | 0.87 | 0.72 | 1.06 | 0.18 |
| Coagulopathy | 1.29 | 1.10 | 1.50 | 0.001 |
| Obesity | 1.04 | 0.91 | 1.19 | 0.54 |
| Weight loss | 0.84 | 0.73 | 0.97 | 0.02 |
| Fluid and electrolyte disorders | 0.79 | 0.69 | 0.91 | 0.001 |
| Blood loss anemia | 1.16 | 0.91 | 1.48 | 0.22 |
| Deficiency anemia | 0.86 | 0.72 | 1.02 | 0.08 |
| Psychoses | 0.64 | 0.52 | 0.78 | <0.001 |
| Depression | 0.66 | 0.59 | 0.74 | <0.001 |
| MEWS Score^a^ | 1.73 | 1.69 | 1.78 | <0.001 |
| Alcohol only | 1.63 | 1.33 | 2.01 | <0.001 |
| Opioid only | 1.33 | 1.05 | 1.70 | 0.02 |
| Polysubstance | 1.76 | 1.42 | 2.20 | <0.001 |
| ADI State Rank^b^ | 1.12 | 1.10 | 1.15 | <0.001 |

^a^MEWS = Modified Early Warning Score; ^b^ADI = Area Deprivation Index;

Supplementary Table F. Fully Adjusted Model for Length of Stay

|  | Estimate | Std. Error | P-value |
| --- | --- | --- | --- |
|  |  |  |  |
| >=1 EMS visit | 2.06 | 0.26 | <0.001 |
| Age | 0.08 | 0.00 | <0.001 |
| Female | -0.41 | 0.12 | 0.001 |
| Congestive heart failure | 0.47 | 0.23 | 0.04 |
| Cardiac arrhythmias | -0.66 | 0.15 | <0.001 |
| Valvular disease | 0.52 | 0.21 | 0.01 |
| Pulmonary circulation disorders | 0.24 | 0.23 | 0.29 |
| Peripheral vascular disorders | 0.45 | 0.19 | 0.02 |
| Hypertension, uncomplicated; | -0.32 | 0.16 | 0.04 |
| Hypertension, complicated | -0.16 | 0.25 | 0.51 |
| Paralysis | 0.47 | 0.35 | 0.18 |
| Other neurological disorders | 0.60 | 0.16 | <0.001 |
| Chronic pulmonary disease | -0.27 | 0.14 | 0.05 |
| Diabetes, uncomplicated | 0.02 | 0.25 | 0.94 |
| Diabetes, complicated | 0.35 | 0.29 | 0.23 |
| Hypothyroidism | -0.40 | 0.20 | 0.05 |
| Renal failure | 0.92 | 0.24 | <0.001 |
| Liver disease | 0.22 | 0.15 | 0.14 |
| Peptic ulcer disease, excluding bleeding | 0.07 | 0.24 | 0.78 |
| AIDS/HIV | -1.83 | 0.59 | 0.002 |
| Lymphoma | 0.64 | 0.50 | 0.20 |
| Metastatic cancer | 0.24 | 0.33 | 0.47 |
| Solid tumor, without metastasis | 0.41 | 0.23 | 0.07 |
| Rheumatoid arthritis/collagen vascular disease | -0.37 | 0.22 | 0.10 |
| Coagulopathy | 0.88 | 0.19 | <0.001 |
| Obesity | 0.07 | 0.16 | 0.68 |
| Weight loss | 0.36 | 0.16 | 0.03 |
| Fluid and electrolyte disorders | -0.49 | 0.16 | 0.002 |
| Blood loss anemia | 0.45 | 0.30 | 0.14 |
| Deficiency anemia | -0.02 | 0.20 | 0.94 |
| Psychoses | -0.39 | 0.20 | 0.06 |
| Depression | -1.01 | 0.13 | <0.001 |
| MEWS Score^a^ | 0.71 | 0.04 | <0.001 |
| Alcohol only | 0.02 | 0.20 | 0.94 |
| Opioid only | 1.12 | 0.24 | <0.001 |
| Polysubstance | 0.78 | 0.21 | <0.001 |
| ADI State Rank^b^ | 0.30 | 0.02 | <0.001 |

^a^MEWS = Modified Early Warning Score; ^b^ADI = Area Deprivation Index;
